# Medical Register

**Published:** 1873-04-01

**Authors:** S. W. Butler

**Affiliations:** 115 S. Seventh street, Philadelphia, Pa.


### MEDICAL REGISTER.

We call the attention of our readers to the following notice, and hope every member of the profession will promptly respond to such circulars as may be received, and thereby facilitate the work of Dr. Butler. The work is one involving much labor on his part, and will be of great value to the profession. Every facility should be given for its early appearance :

MEDICAL REGISTER AND DIRECTORY OF THE UNITED STATES.—Work on this important publication, which was delayed by illness, is now resumed with energy, and it will be issued as soon as the vast amount of material can be collected and arranged. The *Register and Directory* will contain as complete a list of the medical men in the United States, with their professional status, as can be obtained by personal application to each, and from other sources. Also, a complete medical history of each State, including all its medical institutions, societies, etc., and all medical legislation. Nothing will be omitted that will be of possible benefit to the profession. The co-operation of medical men in every section of the country is earnestly solicited in replying to the circulars sent out, and in *giving brief outlines of the history of medical institutions, hospitals, colleges, etc., and of State, and the more important local medical organizations.*

Circulars will be sent out hereafter *by States*, and announced through the medical journals. They *have been sent* to the following States and Territories, viz.: Alabama, Arizona, Arkansas, California, Colorado, Connecticut, Dakotah, Delaware, District of Columbia, Florida, and Georgia. *In March* they will be sent to Illinois, Indiana, Iowa, Kansas, Kentucky, and Louisiana, and all the remaining Territories. IMMEDIATE ANSWERS are requested, and prompt notification in circulars are not received.

Physicians are particularly requested to make as full replies as possible to the questions in the circular.

*Medical Journals please notice.*

S. W. BUTLER, M.D.

115 S. Seventh street, Philadelphia, Pa.

